# Evaluation of chemical-specific IgG antibodies in male workers from a urethane foam factory

**DOI:** 10.1186/s12199-018-0713-4

**Published:** 2018-06-19

**Authors:** Mayumi Tsuji, Yasuhiro Ishihara, Toyohi Isse, Chihaya Koriyama, Megumi Yamamoto, Noriaki Kakiuchi, Hsu-Sheng Yu, Masayuki Tanaka, Takuto Tsuchiya, Masanori Ohta, Rie Tanaka, Toshihiro Kawamoto

**Affiliations:** 1 Department of Environment Health, University of Occupational and Environmental Health, Kitakyushu, Japan; 2 Laboratory of Molecular Brain Science, Graduate School of Integrated Arts and Sciences, Hiroshima University, Higashihiroshima, Japan; 3 Department of Epidemiology and Preventive Medicine, Kagoshima University Graduate School of Medical and Dental Sciences, Kagoshima, Japan; 4 Department of Environment and Public Health, National Institute for Minamata Disease, Minamata, Japan; 5 Institute of industrial and Ecological Sciences, University of occupational and Environmental Health, Kitakyushu, Japan; 6 Department of Food Science, College of Agriculture, National Pingtung University of Science and Technology, Pingtung, Taiwan, Republic of China; 7 Department of Food and Health Sciences, International College of Arts and Sciences, Fukuoka Women’s University, Fukuoka, Japan

**Keywords:** Chemical-specific IgG antibodies, Occupational allergy (OA), Urethane foam factory, Male workers

## Abstract

**Background:**

Plastic resins are complex chemicals that contain toluene diisocyanate (TDI) and/or trimellitic anhydride (TMA), which cause occupational allergies (OA), including respiratory allergies. Serum IgGs against TDI and TMA have been suggested as potential markers of the exposure status and as exploring cause of OA. Although TDI-specific IgG has been examined for suspected OA, TMA-specific IgG is not commonly evaluated in a urethane foam factory. This study therefore investigated both TDI- and TMA-specific IgGs in suspected OA patients and to evaluate the usefulness of the measurement of multiple chemical-specific IgG measurement for practical monitoring.

**Methods:**

Blood samples were collected from two male workers who developed respiratory allergies supposedly caused by occupational exposure to TDI and/or TMA for the presence of TDI- and TMA-specific IgGs. In addition, blood samples from 75 male workers from a urethane foam factory, along with 87 male control subjects, were collected in 2014 and tested for the same IgGs in 2014. The presence and levels of TDI- and TMA-specific serum IgGs were measured using dot blot assays.

**Results:**

We found that controls had mean concentrations of TDI- and TMA-specific IgGs of 0.98 and 2.10 μg/mL, respectively. In the two workers with respiratory allergies, the TDI-specific IgG concentrations were 15.6 and 9.51 μg/mL, and TMA-specific IgG concentrations were 4.56 and 14.4 μg/mL, which are clearly higher than those in controls. Mean concentrations of TDI- and TMA-specific IgGs in the factory workers were 1.89 and 2.41 μg/mL, respectively, and are significantly higher than those of the controls (*P* < 0.001 and *P* < 0.026 for TDI- and TMA-specific IgGs, respectively).

**Conclusion:**

The workers suspected of OA showed an evidently high level of TDI- and TMA-specific IgG, and these levels in workers at the urethane foam factory were also significantly higher than those in controls. In conclusion, the measurement of TDI- and TMA-specific IgG among workers using plastic resins is helpful to monitor their exposure status.

## Background

Plastic products are widely used across the society. Most chemicals employed in the production of widely used plastic products and polymers are known to be hazardous to human health [1]. Toluene diisocyanate (TDI), a polymer of polyurethane resin, is a suspected cause of occupational allergies (OAs) in workers exposed to plastic resins [1, 2]. When a worker dealing with TDI develops symptoms of respiratory allergy, OA is often suspected and tested for by examining the levels of TDI-specific immunoglobulin Gs (IgGs) in the patient’s blood [3, 4]. As reported by Ye et al., TDI exposure levels are positively correlated with the levels of IgGs against TDI-albumin conjugates in workers handling paints and polishes [5].

Plastic resins are mixtures of chemical substances containing curing agents, catalysts, fire retardants, and surfactants in addition to polymers [6]. Trimellitic anhydride (TMA), which is widely used in many industries, is useful as a curing agent, modifier, plasticizer, and surfactant for epoxy and urethane resins [7–9]. Like TDI, TMA is also known to cause respiratory allergies [10, 11], and TMA-specific IgGs have been detected in workers exposed to this chemical [12].

Since allergen removal is the preferred treatment option for patients with allergic diseases, determining causal allergen (s) is a crucial step in helping these patients. However, in most allergies, it is impossible to identify causal allergens through clinical symptoms alone, as most of these symptoms are not allergen-specific. Therefore, detecting the presence of TDI- and TMA-specific IgGs in serum is a very useful method by which TDI- and TMA-related OAs can be identified. However, as of now, only TDI-specific IgGs are used to test for OAs in workers handling urethane resins [2, 13, 14]. However, since TMA has a possibility to be used in conjunction with TDI in many factories, it is also essential to find a marker that can be employed to identify OAs caused by TMA exposure.

The aim of this study is to investigate the levels of TDI- and TMA-specific IgGs in factory workers from a urethane foam factory, as well as suspected OA patients from this site. We also evaluate the usefulness of measuring the levels of chemical-specific IgGs in practical monitoring the exposure of workers to TDI and TMA.

## Methods

### Study subjects

#### Subjects suspected of developing OA

In May 2014, we identified two male workers who exhibited worsening respiratory symptoms especially while working in the urethane foam factory. One patient (worker A) developed symptoms of rhinitis and coughing, while the other patient (worker B) only developed a cough. Both subjects began showing these allergic symptoms from January 2014.

#### Urethane foam factory workers

Blood samples from a total of 75 male workers from a urethane foam factory in Fukuoka, Japan, were obtained for this study in May 2014. The participation rate of workers from this factory was 93%; a total of 77 workers (including the two subjects suspected of OA) out of 83 workers participated in the study. Six workers refused to participate either because they were ill or because they refused to allow blood collection.

#### Control subjects

Blood samples from a total of 87 males under the age of 60 years from Minami-Kyushu City in Japan were collected to serve as controls in July 2014. Among this group, 63 subjects (72%) were local government employees. Although five subjects (6%) in this group were also factory workers, none of these subjects has used or handled plastic resin at the factories.

### Detection of chemical-specific IgGs

#### Serum samples

Approximately 5 mL of blood was collected from all subjects and centrifuged at 3000 rpm for 10 min to obtain serum samples.

#### Detection of chemical-specific IgG by dot blotting

Levels of chemical-specific IgGs were determined using the dot blot assay which the co-author (TK) has developed and patented [15–17]. In this study, the diagnostic antigens were obtained by mixing human serum albumin (Wako Pure Chemical, Japan.) with the resin precursors TDI (Wako) and TMA (Wako) in a 1:100 ratio at pH 10.8. Details of this procedure have been reported elsewhere [15–17].

### Questionnaire survey

Information on smoking and alcohol consumption and allergic history (asthma, allergic rhinitis, atopic dermatitis, allergic conjunctivitis, and contact dermatitis), as well as usage of plastic resin in daily life was obtained through a self-administered questionnaire. In addition, the two patients suspected of developing OA were interviewed by industrial physicians.

### Statistical analyses

Two-group comparisons were performed using the Mann–Whitney *U* test or multivariate logistic regression analyses. Three-group comparisons were performed using the Kruskal–Wallis test. We divided the factory workers and controls into three groups (T1–T3), depending on the levels of chemical-specific IgGs detected in controls, and estimated odds ratio (OR) and corresponding 95% confidence intervals (CIs) after adjusting for the effects of age and allergy history on chemical-specific IgG levels.

All statistical analyses were performed in STATA version 14 (Stata Corp, College Station, TX), and results were considered to be statistically significant at *P* < 0.05 (two-sided).

### Ethics review

This study was approved by the Institutional Ethics Committee at the University of Occupational and Environmental Health in 2013 (H25-008) and Kagoshima University Graduate School of Medical and Dental Sciences in 2014 (486). Written informed consents were obtained from all participants.

## Results

### Age, lifestyle characteristics, and immunoglobulin profiles of patients suspected of developing OA

Table 1 shows age, lifestyle characteristics, and IgG profiles of the two patients suspected of developing OA. Both have worked in the manufacturing department of the urethane foam factory since they were employed. Total IgE, IgG, and TDI-specific IgE levels were measured at a commercial company (SRL, Inc. in Japan), though the TMA-specific IgE test was not commercially available.

**Table 1:** Characteristics and immunoglobulin profiles of the two subjects suspected of developing OA

	Worker A	Worker B
Age (year)	43	52
Employment period (year)	3	27
Smoking habits	No	No
Habitual alcohol drinking	No	No
Allergic history	Allergic rhinitis	Allergic rhinitis
Immunoglobin profile		
TDI-specific IgG (μg/mL)	15.62	9.51
TMA-specific IgG (μg/mL)	4.56	14.39
TDI-specific IgE (UA/mL)	≤ 0.34	≤ 0.34
Total IgG (mg/dL)	1597	1180
Total IgE (IU/mL)	29.4	106

### Characteristics of urethane foam factory workers and control subjects

#### Urethane foam factory workers

The median, 25th percentile, and 75th percentile of workers’ ages were 37.0, 31.0, and 47.0 years, respectively. The percentages of smokers and habitual alcohol drinkers were 48 and 51%, respectively. In this group, 33% of the subjects had a history of allergy. The median employment period was 10 (range = 1–35) years.

#### Control subjects

The median, 25th percentile, and 75th percentile of control subjects’ ages were 49.0, 39.0, and 52.0 years, respectively. The percentage of smokers and habitual alcohol drinkers were 30 and 29%, respectively. In this group, 40% of the subjects had a history of allergy.

### TDI- and TMA-specific IgG concentrations in urethane foam factory workers and controls

Table 2 shows the distribution of TDI- and TMA-specific IgG concentrations in the workers and controls, along with other factors such as age and lifestyle characteristics. Age had a significant effect on the levels of TDI-specific IgGs in the control group (*P* = 0.033). The median values of IgG in the urethane factory worker and control groups were lower than those of the two patients suspected of developing OA. However, there were some workers, and even some controls who had higher TDI- and TMA-specific IgG levels than the two patients, suspected of having developed OA.

**Table 2:** TDI- and TMA-specific IgG concentrations in urethane foam factory workers and controls

Geometric mean of IgG (95% CI) (μg/mL)							
Controls				Urethane foam factory workers			
Number for TDI (TMA)		TDI	TMA	Number	TDI	TMA	P value*
Median of IgG (range)		0.81 (ND–12.9)	1.77 (ND–36.8)		2.07 (ND–29.1)	2.58 (ND–74.6)	< 0.001 (TDI) 0.026 (TMA)
Age (years)							
Below 39	23 (23)	1.22 (0.75, 1.98)	3.26 (2.09, 5.08)	44	1.69 (1.10, 2.59)	2.44 (1.46, 4.07)	
40–49	23 (22)	1.41 (0.91, 2.18)	1.46 (0.64, 3.32)	19	1.91 (0.94, 3.86)	2.33 (1.07, 5.08)	
50 and above	41 (40)	0.64 (0.35, 1.20)	1.92 (1.26, 2.93)	12	2.82 (1.47, 5.42)	2.41 (0.94, 6.19)	
*P* value^#^		0.033	0.173		0.341	0.451	
Median (25 and 75 percentile)		37 (31, 47)			49 (39, 52)		< 0.001
Allergic history							
No	49 (48)	1.19 (0.89, 1.61)	2.01 (1.39, 2.91)	50	1.95 (1.33, 2.85)	2.24 (1.36, 3.71)	
Yes	38 (37)	0.75 (0.40, 1.42)	2.22 (1.29, 3.80)	25	1.79 (1.00, 3.20)	2.78 (1.64, 4.71)	
*P* value^$^		0.686	0.716		0.800	0.757	
Smoking habit**							
No	61 (60)	0.95 (0.70, 1.29)	1.83 (1.23, 2.71)	39	1.76 (1.09, 2.82)	2.12 (1.35, 3.34)	
Yes	26 (25)	1.06 (0.46, 2.47)	2.90 (1.87, 4.49)	36	2.07 (1.38, 3.12)	2.75 (1.48, 5.12)	
*P* value^#^		0.496	0.195		0.668	0.311	
Habitual alcohol drinking**							
No	62 (61)	0.90 (0.58, 1.39)	2.15 (1.47, 3.14)	37	1.80 (1.05, 3.06)	3.24 (2.02, 5.20)	
Yes	25 (24)	1.19 (0.83, 1.71)	1.99 (1.16, 3.43)	38	2.00 (1.41, 2.84)	1.82 (1.02, 3.24)	
*P* value^$^		0.262	0.577		0.678	0.145	

*ND* not detected

^#^*P* values were obtained using Kruskal–Wallis test

^$^P values were obtained using Mann–Whitney *U* test

*P values were calculated for the comparison between controls and factory workers by Mann–Whitney *U* test

**P values were less than 0.05 in comparison of the prevalence of smokers and alcohol drinkers between controls and factory workers

There were no workers who used plastic resin in daily life, but there was only one control who used plastic resin in daily life. However, TDI- and TMA-specific IgGs were not detected in this control.

### Differences in the distribution of TDI- and TMA-specific IgG levels between urethane foam factory workers and controls

**Fig. 1:**
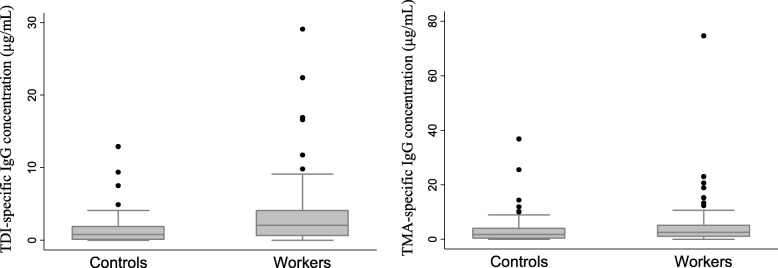
The distribution of TDI- and TMA-specific IgGs in the urethane foam factory workers and controls

Among the control group, the geometric means of TDI- and TMA-specific IgGs were 0.98 μg/mL (0.72, 1.34; 95% CI) and 2.10 μg/mL (1.55, 2.84; 95% CI), respectively. TMA-specific IgG could not be measured for the two control subjects because of the shortage of the antigen whose antigenicity should be identical with that used for workers’ IgG measurement. The geometric means of TDI- and TMA-specific IgGs for the urethane foam factory workers were 1.89 μg/mL (1.39, 2.59, 95% CI) and 2.41 μg/mL (1.66, 3.50; 95% CI), respectively. The mean concentrations of TDI- and TMA-specific IgGs in the factory workers were significantly higher than those of controls (*P* < 0.001 and *P* = 0.026, respectively) by Mann–Whitney *U* test (Fig. 1).

The geometric means of TDI- and TMA-specific IgGs for controls were 0.98 μg/mL (95% CI 0.72, 1.34) and 2.10 μg/mL (95% CI 1.55, 2.84), respectively. The geometric means of TDI- and TMA-specific IgGs for the urethane foam factory workers were 1.89 μg/mL (95% CI: 1.39, 2.59) and 2.41 μg/mL (95% CI: 1.66, 3.50), respectively. The mean concentrations of TDI- and TMA-specific IgGs in the factory workers were significantly higher than those of controls (*P* < 0.001 and *P* = 0.026, respectively) as determined by Mann–Whitney *U* test.

Logistic regression analyses also show that the prevalence of factory workers in the T3 category in the TDI-specific IgG group was significantly higher than that of controls (OR 2.71; 95% CI 1.22, 6.04) (Table 3), indicating that more factory workers had high levels of TDI-specific IgG than controls. Although the number of factory workers having high values of TMA-specific IgGs was also more than those of controls, this difference was not found to be significant.

**Table 3:** The comparison of TDI- and TMA-specific IgGs between the urethane foam factory workers and controls

Concentration of specific IgG^‡^	Controls	Workers	OR (95% CI)	*P* value^†^
TDI (μg/mL)	*N* = 87	*N* = 75		
T1 (< 0.47)	29	16	1.00 (reference)	
T2 (≥ 0.47 < 1.28)	29	12	0.71 (0.27–1.84)	0.479
T3 (≥ 1.28)	29	47	2.71 (1.22–6.04)	0.015
			*P* for trend = 0.002	
TMA (μg/mL)	*N* = 85	*N* = 75		
T1 (< 1.00)	28	14	1.00 (reference)	
T2 (≥ 1.00 < 3.00)	28	24	1.50 (0.62–3.60)	0.369
T3 (≥ 3.00)	29	37	2.14 (0.92–4.98)	0.078
			*P* for trend =0.060	

^†^OR, 95% CI, and *P* values were obtained using multivariate logistic regression analysis adjusted for the effects of age and allergic history

^‡^Concentration of specific IgGs were divided into three groups (T1–T3) depending on the levels of chemical-specific IgGs detected in controls

## Discussion

This study reveals that workers who handle plastic resins in urethane foam factories are likely to have significantly higher levels of TDI- and TMA-specific IgGs in their blood serum than in control subjects who do not handle plastic resins. Furthermore, we find that workers who develop OAs have higher TDI- and TMA-specific IgGs than controls. These findings indicate that measuring the levels of chemical-specific IgGs may be helpful in evaluating if workers handling plastic resins have been exposed to TDI and TMA, and if they have developed OAs against these chemicals.

In this study, two workers (labeled A and B), who developed clinical symptoms of OA, were found to have high levels of TDI- and TMA-specific IgGs. According to an interview with industrial physicians, some of the workers might not wear a protective equipment properly, which could affect the exposure level for each worker. However, these workers’ IgG levels were not always higher than those observed in other workers without OA symptoms; furthermore, a few samples from the control group also have high levels of TDI- and TMA-specific IgGs. These findings indicate that there can be a lot of variation in an individual’s susceptibility to OA and that, although high levels of TDI- and TMA-specific IgGs may serve as indicators of chemical exposure, they may not be very accurate diagnostic indicators of OA development. In addition, due to wide variations in the levels of TDI- and TMA-specific IgGs among individuals, monitoring each factory worker individually over different periods of time with reference to the Japanese Guidelines for Diagnosis and Management of Occupational Allergic Diseases (2016) may be necessary for OA diagnosis. In addition, personal monitoring of chemical-specific IgG will be helpful to evaluate the individual exposure status after the employment.

In order to reduce the symptoms or prevent the development of OA, prompt evaluations of a worker’s exposure status to possible allergens are crucial. Since workers handling urethane resins are likely to be exposed to many chemical allergens, monitoring the levels of multiple chemical-specific IgGs in their blood simultaneously is a practical and efficient method to keep track of their exposure status to various chemicals. The dot blot assay used in this study is a very practical technique with which multiple chemical-specific IgGs can be evaluated simultaneously in a short period of time.

In this factory, TDI concentration has never exceeded the recommended occupational exposure levels (0.005 ppm) [18] for the last decade. Since the factory is under no legal obligation to measure or maintain TMA levels, there were no records for TMA concentrations within the factory premises. According to the report by Ghosh et. al [12], TMA-specific IgG is useful not only as an indicator of exposure but also as a predictor of subsequent onset of OA when a high TMA-specific IgG is detected. In this study, one factory worker, without allergic symptoms, showed a high level of TMA-specific IgG (74.6 μg/mL), indicating at a potential risk of OA. The detailed monitoring of the levels of both TDI and TMA will be important and necessary to maintain occupational health.

There are some limitations in our study. First, our study also found that TDI- and TMA-specific IgGs could be present in the control group, who were recruited from Minami-Kyushu City in Japan; this is in agreement with several studies that report the occurrence of chemical-specific IgGs in the general population [5, 19, 20]. Therefore, for employees already showing high chemical-specific IgG levels before occupational exposure, special considerations are required including job assignment with no/low chemical exposure and regular monitoring of chemical-specific IgG. Second, there are no defined cut-off points of TDI- and TMA-specific IgGs for diagnosis of OA and exposure. Further studies are required to understand the clinical significance of these findings.

## Conclusion

The workers suspected of OA showed an evidently high level of TDI- and TMA-specific IgG, and these levels among workers at the urethane foam factory were also significantly higher than those in controls. In conclusion, the measurement of TDI- and TMA-specific IgG levels among workers handling plastic resins maybe helpful in monitoring their exposure status.

## Abbreviations

OA: Occupational allergy
TDI: Toluene diisocyanate
TMA: Trimellitic anhydride
